# Low-research settings: is there a need for specific attention from funders?

**DOI:** 10.1136/bmjgh-2024-017226

**Published:** 2025-09-11

**Authors:** Sahani de Silva, Mike English, Paul Newton

**Affiliations:** 1 School of Medical and Biomedical Sciences, University of Oxford, Oxford, UK; 2 Health Systems Collaborative, Nuffield Department of Medicine Centre for Global Health Research, University of Oxford, Oxford, UK; 3 Health Services Unit, KEMRI-Wellcome Trust Research Programme, Nairobi, Kenya; 4 Centre for Tropical Medicine and Global Health, University of Oxford Nuffield Department of Medicine, Oxford, UK; 5 Mahidol Oxford Tropical Medicine Research Unit, Bangkok, Thailand

**Keywords:** Global Health, Health policies and all other topics

## Abstract

In order to achieve global equity in healthcare research it is important to identify countries that are less visible in this regard, to inform interventions. We performed a bibliometric analysis of the Web of Science Core Collection to identify countries with less visible medical research output, particularly in the English language. This highlighted that an important distinction needs to be made between low-resource and low-research countries, as countries of diverse World Bank income classifications and population size appeared as low-research countries in our search. We discuss potential contributors to these inequalities and implications for potential funders and collaborators. Owing to the English language bias of the database used for our search, this piece is aimed, in particular, at Anglophone institutions. It highlights potential issues of coloniality in the healthcare research landscape and provides suggestions to address research equity through more active engagement with countries.

**Summary box**

- We performed a bibliometric analysis of global medical research output between 2011 and 2022 to identify countries that are less represented in this regard, with research outputs that may least reflect national and regional health concerns in the predominantly English-language scientific arena.
- The results demonstrate a need to distinguish between low-resource, and low-research countries that should perhaps be prioritised for funding and collaboration to extend participation in research at a global level.
- We highlight potential barriers to research visibility affecting low-research countries and the role of coloniality in driving this inequality. We discuss implications of these findings for potential funders and collaborators, particularly from Anglophone institutions, and urge them to actively engage with these countries to promote equity in global research, while working to restructure the systems that disadvantage them.

## Introduction

 Research can shape the success of a country’s healthcare system. Medical research must investigate not only laboratory and clinical outcomes, but also the structure of healthcare systems and the behavioural, social and economic factors affecting them.^1^ This underpins the need for local research that considers a country’s unique healthcare landscape to promote health and address its disease burden. This is becoming increasingly important in the context of climate change, which is having non-uniform effects on health patterns globally, disproportionately affecting lower-middle-income countries (LMIC).^2 3^ Publications are one measurable, albeit imperfect, output of scientific research.^4^ To support research, there is a welcome shift in opportunities that should enable more LMIC investigators to access support from wealthier countries’ funding institutions. However, eligibility or success in accessing this may depend on perceived credibility and research track record of LMIC investigators and parent institutions. This may produce a ‘reverse-Matthew effect’, where those unable to demonstrate research potential through publication, may have difficulty obtaining the funding that should strengthen capabilities. We therefore sought to identify which countries are least visible, assessed using health research publication output. Using Web of Science (WoS) biases these results towards English-language output,^5^ so potentially this is a more specific metric of research visibility to funders from Anglophone regions. This piece therefore aims to highlight issues of coloniality potentially disadvantaging countries in the current research landscape, particularly when seeking funding from UK and US institutions, underpinning the need for such funders to actively seek and engage with these countries to promote research equity.

## A bibliometric analysis of the WoS database

We performed a bibliometric analysis of health research output globally from 2011 to 2022, modifying the methods of Badenhorst and colleagues to encompass a broader definition of medical research beyond public health^6^ (online supplemental document). We extracted the number of results per year for each country based on any listed author reporting an affiliation with an institution in that country. When comparing trends over time, years were grouped into three-year blocks to minimise stochastic variation (2011–2013, 2014–2016, 2017–2019 and 2020–2022).^6^ We included largely the same countries and regional classification in our search as Badenhorst and colleagues^6^ (online supplemental document).

**Figure 1:**
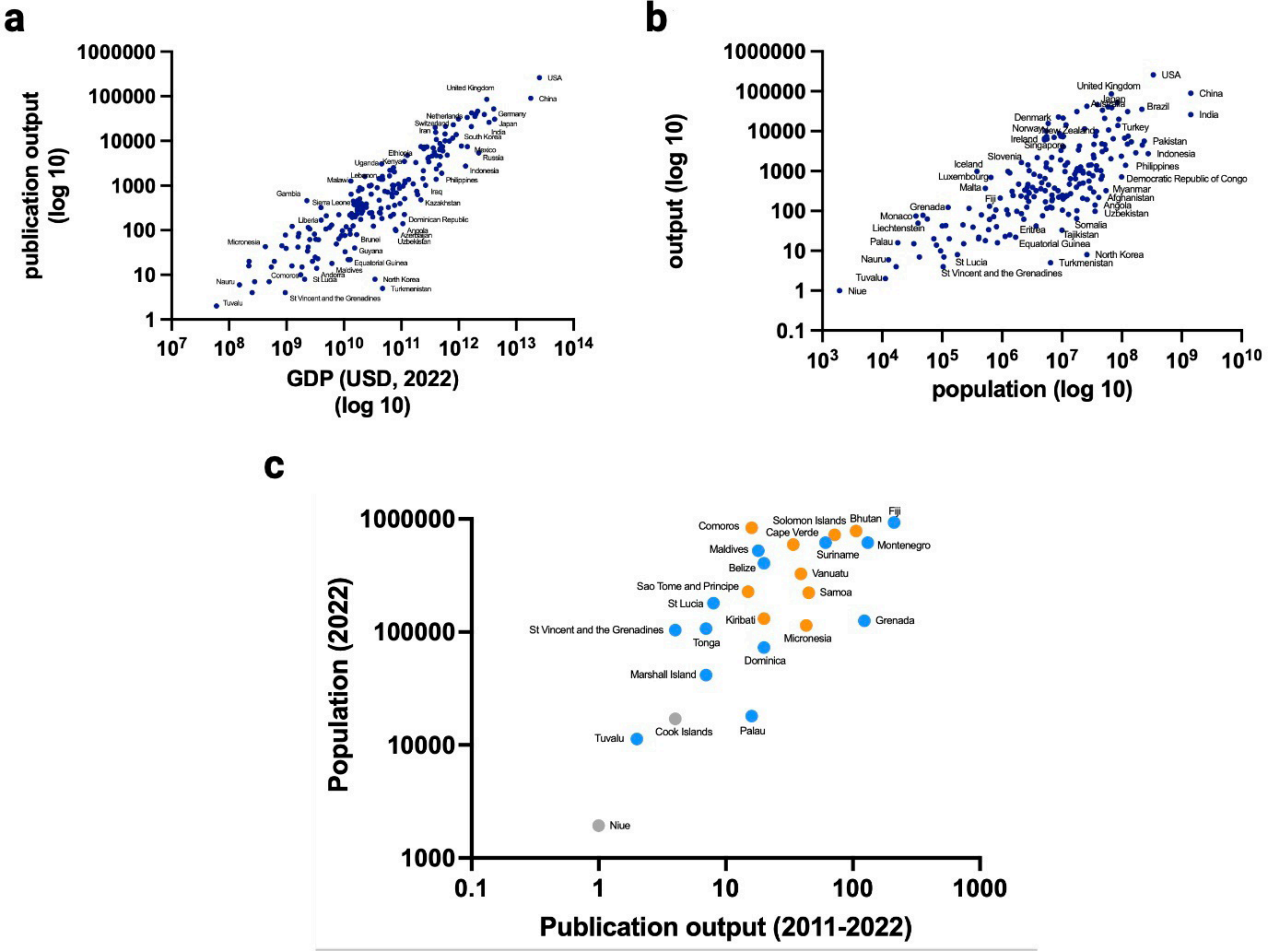
*Initial analysis*. (**a**) Scatter plot of publication output (2011–2022) against gross domestic product (GDP) in USD (2022) (**b**) Scatter plot of publication output (2011–2022) against population (2022) (**c**) Very small countries (population <1M) by population (2011–2022) with World Bank income classification excluding high-income countries. Orange=lower-middle-income countries, blue=upper-middle-income countries, grey=unclassified.

When comparing 2011–2013 with 2020–2022, publication output increased, both globally and individually for all 199 countries in our search. We focused on the relationship between output and a country’s gross domestic product, as well as its population. Unsurprisingly, output correlated with both (figure 1a and b). We then classified countries by income and population size. For the latter, we classified countries as very small (population < 1 million), small (1 million < population < 10 million), moderate (10 million < population < 100 million) or large (population > 100 million). We used World Bank income classification and excluded high-income countries from further analyses as we amed to identify countries with low research visibility, possibly being neglected by funders.

## Low-resource settings versus low-research settings; an important distinction

Many very small countries occupied the lower places after ranking all countries by total output. Within this subset, the relationship between population and publication output remained strong (figure 1c, online supplemental document).

**Figure 2:**
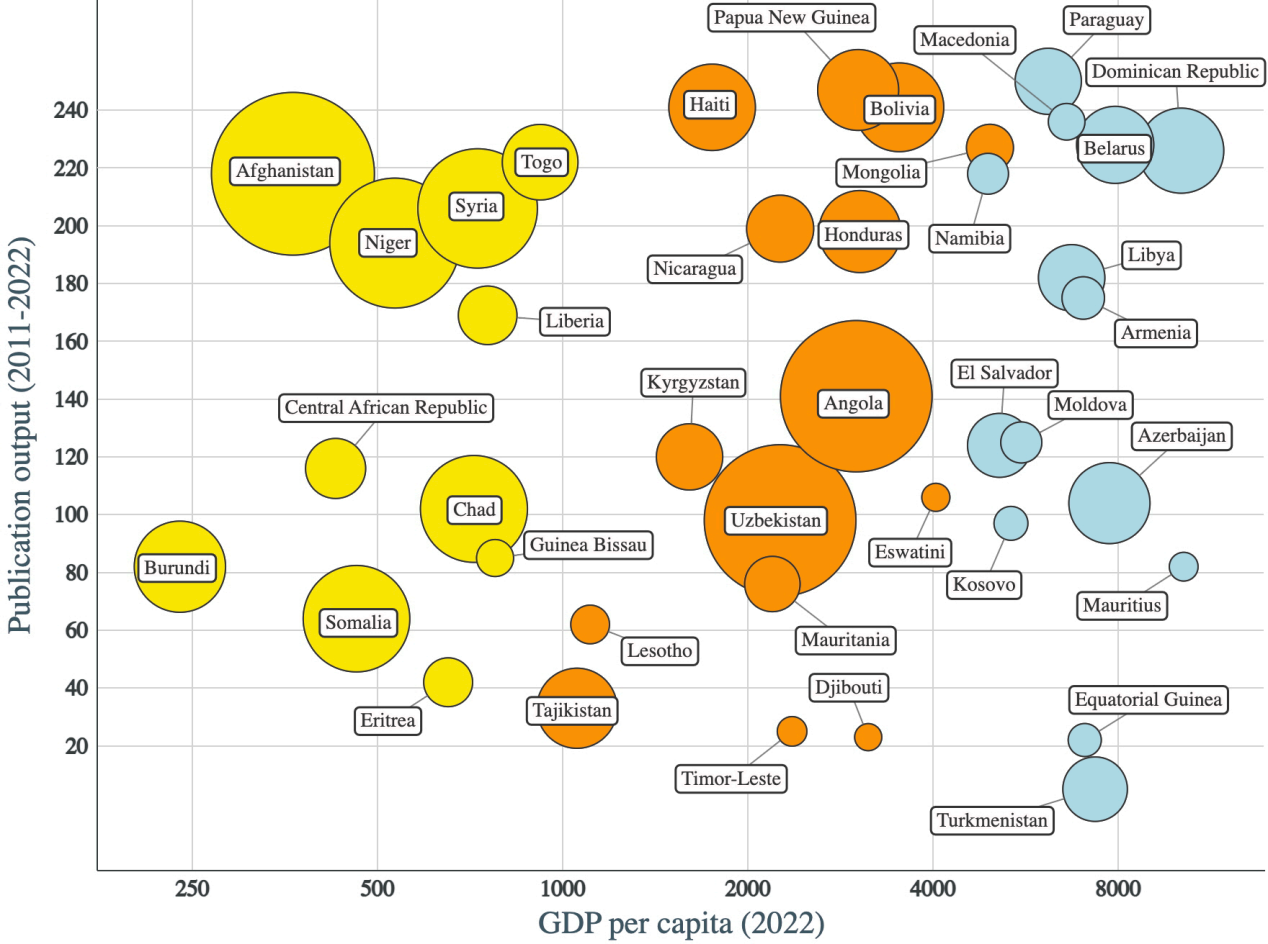
*Low-research settings*. Bubble plot of 40 lowest countries in terms of publication output against gross domestic product (GDP) per capita (USD, 2022), excluding high-income countries and very small countries. Bubble colour represents World Bank income classification and bubble size represents country population (2022). Yellow=low-income countries, orange=lower-middle-income countries, blue=upper-middle-income countries.

After excluding very small countries, we identified 73 countries with total publication output <1000 between 2011 and 2022 (online supplemental document). Importantly, this metric serves as a measurement of research visibility. Countries with poorer visibility are hereon referred to as low-research countries for brevity. These were diverse in size and income classification; countries such as Benin, the Democratic Republic of Congo, Sudan and Venezuela appeared as moderately-sized LMIC with lower output. We then focused on the 40 countries with the lowest total output after excluding very small countries. Although North Korea was within this group, we excluded it from further analysis given its political isolation and ineligibility for support from many Western nations. This list of 40 still included countries of differing income classification and both small and moderate size (figure 2). Subsequent analysis of publication output per capita demonstrated that this list remained largely similar, with some exceptions (See online supplemental document).

These findings highlight the importance of a more pragmatic approach to informing allocation of research support. Underlying drivers of research capacity and production are often not singular determinants such as income status or population, but rather an interaction of multiple systemic issues that change over time. To acknowledge this, a distinction must be made between ‘low-resource’ and ‘low-research’ settings. For example, Bolivia, the Dominican Republic and Honduras emerge as low-research countries despite their larger size and higher income classification, while Guinea Bissau appears as a higher performer for its population and income status, no longer being represented in the lowest 40 when publication output is adjusted for population size. This distinction may serve as an important tool for unbiased identification of low-research settings.

Due to the heterogeneity of World Bank income classification groups in the 40 low-research countries highlighted, we sought to identify potential additional barriers to research and publication. It is likely that structural factors such as political climate play an important role. For example, Turkmenistan, which ranks lowest within this list, has been under totalitarian rule since independence. Other countries including Afghanistan, Eritrea and Syria have a history of significant conflict. In fact, many countries within this list rank poorly when using more formal measures of good governance.^7^

Language is likely to be another important contributor to this disparity. Africa and central and southern America were highly represented in this list. Notably, a number of these African countries are historically Franco or Luso-phone, for example Burundi, Chad and Angola, while in central and southern America, Spanish is widely spoken. In fact, excluding Eritrea, Liberia, Mauritius and Namibia, English is not declared as an official or working language in any other of the 40 low-research countries highlighted.^8^ This is a limitation of our analysis as WoS is highly biased towards English-language journals and therefore likely to under-represent research outputs if English is not the main language of publication.^5 6^

This English-language bias is reported to under-represent output from Russia in stroke research, and Man and colleagues identified a relationship between medical publication output in highly-ranked journals and English proficiency in high-income countries.^9 10^ This may explain why seven of the nine countries belonging to the Europe region within our list of 40 low-research countries are former Soviet republics. The inherent bias of categorising countries based on English-language publications is important to acknowledge. However, as a measure of ‘visibility’ of research outputs to British and American funders, this metric may be influential. Scientists and institutions that are less likely to publish in English may find it more difficult to demonstrate research credibility to Anglophone funding organisations.

More general barriers to research and publication have also been identified. Importantly, high costs associated with accessing and publishing in many high-impact journals, although being addressed, may still act to prevent equitable research participation. Spending on publication charges may reduce the availability of funds for the actual research, even in a time when sharing knowledge online should be easier.^11^

## Implications for funders and collaborators

We highlight a potential ‘output trap’ where low research outputs contribute to funding challenges which sustain low research outputs in turn. This may perpetuate inequities unless informed interventions are deployed to break the cycle. This requires a greater focus on equity in the identification of countries for resource allocation and collaboration, and suggests a value of distinguishing ‘low-research’ from ‘low-resource’ settings in dialogues surrounding the allocation of resources for supporting medical research globally. We therefore urge potential funders and collaborators to acknowledge this distinction and the power they wield in the research space, the context-specific nature of research gaps and the need for tailored support as a result.^12^

Implications may entail actively focusing some support to low-research countries (even if classified as upper-middle-income). It also emphasises the importance of longer-term capacity strengthening at individual, institutional and systemic levels in funding initiatives. An example of its value may be Guinea-Bissau, which appears to be a high performer for its size and income classification, likely linked to the long-standing collaborative Bandim Health Project.^13^

These disparities also highlight issues of coloniality that are still prevalent in the global research space. The English-language bias in science, funding disparities and scientific gatekeeping affecting research visibility all stem from power imbalances and structural inequalities that are rooted in a history of inequitable relationships that continue to shape health research practice.^14^ Khan argues that tackling this epistemic injustice will require the stepwise decolonisation of knowledge itself and highlights the role that academic journals can play in this process.^15^ He calls for the restructuring of evidence hierarchies as well as a more context-based assignment of credibility to evidence. The aim is to provide a platform for alternative methods of knowledge generation and analysis, beyond those which are considered acceptable from a Eurocentric perspective and perpetuate an over-representation of interests of stakeholders from the Global North. Decolonisation of the research space may also involve investments to improve the quality, reach, open access and indexing of journals from the Global South to diversify global knowledge and raise its visibility.^16^ This may need to go hand-in-hand with addressing language barriers and biases, including within established research publication databases.

Countries with a population <1 million likely represent a unique challenge in healthcare research. Some LMIC are very small and consequently may be unable to sustain an entirely independent research environment. The establishment of research networks among neighbouring small island states is in some cases facilitating collaboration. For example, the Eastern Caribbean Health Outcomes Research Network (ECHORN) and similar models may ensure that even the smallest countries can address their local research needs.^17^

There are important limitations to our analysis. Although we could examine the specificity of our searches, we had no way of assessing their sensitivity. We are likely to have missed some relevant articles published between 2011 and 2022 in our analysis. Although the use of author affiliations as a means of classifying publications by country serves as a good starting point to estimate a country’s research output, the relevance of the research to the author’s country of affiliation would depend on the topic and the author’s role in the work. It would therefore be important to further investigate publications listed under low-research countries in particular. Additionally, the use of author affiliation may overestimate true contributions to research and may risk over-representation of certain countries due to ‘dummy affiliations’.^6 18^ Using the WoS Core Collection that only includes journals with regularly-assessed quality, resulting in many national and non-English language journals being excluded, may disproportionately affect measured output from many LMIC. However, it could also be argued that our results point to the ‘visibility’ of research to Anglophone funding organisations. Finally, the search also risks not including the widespread grey literature that may be locally important for informing healthcare decision-making.

## Conclusion

In conclusion, we conducted a bibliometric analysis of predominantly English-language global medical research output between 2011 and 2022, to identify low-research countries. A country’s income status and population are general predictors of research output as assessed through the WoS. However, there is considerable disparity in research output among countries when grouped by population size and income group, suggesting the presence of low-research countries. These may be disadvantaged when applying to funders, especially those from Anglophone countries, because of challenges scientists and even whole institutions face in demonstrating their scientific credentials. Future work should examine if the award of major national and international grants in recent years demonstrates the same patterns. Funding agencies, especially those in the UK and US, should likely consider longer-term strategies to mitigate barriers to research and publication in low-research settings while acting with awareness of power imbalances that influence this disparity.

## Supplementary material

10.1136/bmjgh-2024-017226 online supplemental file 1

## Data Availability

All publication data extracted from Web of Science Core Collection accessed through the University of Oxford. Raw data files can be provided upon request.
