# Supplementary material for: Low-research settings: is there a need for specific attention from funders?
Source: BMJ Glob Health. 2025 Sep 11;10(9):e017226. doi: 10.1136/bmjgh-2024-017226 (PMC12519391; doi:10.1136/bmjgh-2024-017226)
Supplement: online supplemental file 1 [file bmjgh-10-9-s001.docx]

**Supplementary information**

Countries included in search with regional classification and total output

| **Region** | **Country** | **Total output (2011-2022)** |
| --- | --- | --- |
| Africa | Algeria | 629 |
|  | Angola | 141 |
|  | Benin | 467 |
|  | Botswana | 394 |
|  | Burkina Faso | 892 |
|  | Burundi | 82 |
|  | Cameroon | 1393 |
|  | Cape Verde | 34 |
|  | Central African Republic | 116 |
|  | Chad | 102 |
|  | Comoros | 16 |
|  | Cote d’Ivoire | 557 |
|  | Republic of Congo | 450 |
|  | Democratic Republic of Congo | 714 |
|  | Equatorial Guinea | 22 |
|  | Eritrea | 42 |
|  | Eswatini | 106 |
|  | Ethiopia | 4742 |
|  | Gabon | 333 |
|  | Gambia | 462 |
|  | Ghana | 2525 |
|  | Guinea | 270 |
|  | Guinea Bissau | 85 |
|  | Kenya | 3480 |
|  | Lesotho | 62 |
|  | Liberia | 169 |
|  | Madagascar | 376 |
|  | Malawi | 1272 |
|  | Mali | 503 |
|  | Mauritania | 76 |
|  | Mauritius | 82 |
|  | Mozambique | 767 |
|  | Namibia | 218 |
|  | Niger | 194 |
|  | Nigeria | 4502 |
|  | Rwanda | 641 |
|  | Sao Tome and Principe | 15 |
|  | Senegal | 984 |
|  | Seychelles | 42 |
|  | Sierra Leone | 323 |
|  | South Africa | 10751 |
|  | Togo | 222 |
|  | Uganda | 3061 |
|  | Tanzania | 2025 |
|  | Zambia | 1018 |
|  | Zimbabwe | 838 |
| West Pacific I | Australia | 42319 |
|  | Japan | 30847 |
|  | Singapore | 6322 |
|  | New Zealand | 6635 |
| West Pacific II | Brunei | 80 |
|  | Cambodia | 640 |
|  | China | 90212 |
|  | Cook Islands | 4 |
|  | Fiji | 211 |
|  | Hong Kong | 6963 |
|  | Indonesia | 2735 |
|  | Kiribati | 20 |
|  | Laos | 373 |
|  | Malaysia | 4678 |
|  | Marshall Island | 7 |
|  | Micronesia | 43 |
|  | Mongolia | 227 |
|  | Nauru | 6 |
|  | Niue | 1 |
|  | Palau | 16 |
|  | Papua New Guinea | 247 |
|  | Philippines | 1390 |
|  | South Korea | 21078 |
|  | Samoa | 45 |
|  | Solomon Islands | 72 |
|  | Tonga | 7 |
|  | Tuvalu | 2 |
|  | Vanuatu | 39 |
|  | Vietnam | 2348 |
| America I | Canada | 46596 |
|  | Cuba | 975 |
|  | USA | 260065 |
| America II | Argentina | 4801 |
|  | Bahamas | 22 |
|  | Barbados | 116 |
|  | Belize | 20 |
|  | Bolivia | 241 |
|  | Brazil | 35289 |
|  | Chile | 4275 |
|  | Colombia | 4581 |
|  | Costa Rica | 648 |
|  | Dominica | 20 |
|  | Dominican Republic | 226 |
|  | Ecuador | 1158 |
|  | El Salvador | 124 |
|  | Grenada | 123 |
|  | Guatemala | 395 |
|  | Guyana | 40 |
|  | Haiti | 241 |
|  | Honduras | 198 |
|  | Jamaica | 277 |
|  | Mexico | 7498 |
|  | Nicaragua | 199 |
|  | Panama | 359 |
|  | Paraguay | 250 |
|  | Peru | 2144 |
|  | St Kitts and Nevis | 78 |
|  | St Lucia | 8 |
|  | St Vincent and the Grenadines | 4 |
|  | Suriname | 61 |
|  | Trinidad and Tobago | 244 |
|  | Uruguay | 823 |
|  | Venezuela | 867 |
| South and South-East Asia | Bangladesh | 2893 |
|  | Bhutan | 106 |
|  | India | 26136 |
|  | Maldives | 18 |
|  | Myanmar | 324 |
|  | Nepal | 1429 |
|  | North Korea | 8 |
|  | Sri Lanka | 1006 |
|  | Thailand | 6409 |
|  | Timor-Leste | 25 |
| East Mediterranean | Afghanistan | 218 |
|  | Bahrain | 309 |
|  | Djibouti | 23 |
|  | Egypt | 6729 |
|  | Iran | 20041 |
|  | Iraq | 1023 |
|  | Jordan | 1395 |
|  | Kuwait | 742 |
|  | Lebanon | 1592 |
|  | Libya | 182 |
|  | Morocco | 1367 |
|  | Oman | 507 |
|  | Pakistan | 5640 |
|  | Qatar | 1429 |
|  | Saudi Arabia | 7731 |
|  | Somalia | 64 |
|  | Sudan | 749 |
|  | Syria | 206 |
|  | Tunisia | 1608 |
|  | United Arab Emirates | 1900 |
|  | Yemen | 297 |
| Europe | Albania | 295 |
|  | Andorra | 14 |
|  | Armenia | 175 |
|  | Austria | 8733 |
|  | Azerbaijan | 104 |
|  | Belarus | 228 |
|  | Belgium | 14362 |
|  | Bosnia | 387 |
|  | Bulgaria | 1079 |
|  | Croatia | 2168 |
|  | Cyprus | 985 |
|  | Czech Republic | 4062 |
|  | Denmark | 15656 |
|  | Estonia | 900 |
|  | Finland | 7350 |
|  | France | 39166 |
|  | Georgia | 324 |
|  | Germany | 51947 |
|  | Greece | 7484 |
|  | Greenland | 63 |
|  | Hungary | 3301 |
|  | Iceland | 989 |
|  | Ireland | 5996 |
|  | Israel | 7466 |
|  | Italy | 40453 |
|  | Kazakhstan | 479 |
|  | Kyrgyzstan | 120 |
|  | Latvia | 468 |
|  | Liechtenstein | 49 |
|  | Lithuania | 1021 |
|  | Luxembourg | 691 |
|  | Macedonia | 236 |
|  | Malta | 368 |
|  | Moldova | 125 |
|  | Monaco | 75 |
|  | Montenegro | 131 |
|  | Netherlands | 30916 |
|  | Norway | 10008 |
|  | Poland | 9977 |
|  | Portugal | 7274 |
|  | Romania | 3408 |
|  | Russia | 5392 |
|  | San Marino | 15 |
|  | Serbia | 2199 |
|  | Slovakia | 1260 |
|  | Slovenia | 1650 |
|  | Spain | 33504 |
|  | Sweden | 21310 |
|  | Switzerland | 22947 |
|  | Tajikistan | 33 |
|  | Turkey | 13832 |
|  | Turkmenistan | 5 |
|  | Ukraine | 1088 |
|  | United Kingdom | 85930 |
|  | Uzbekistan | 98 |
| Disputed territories | Taiwan | 11540 |
|  | Kosovo | 97 |
|  | Palestine | 324 |

Extended methods

We searched the Web of Science Core Collection, using the search terms “public health*”, “community health*”, “epidemiol*”, “clinical trial”, “clinical research” and “pathophysiol*”. The search was refined by including publications released from 2011 to 2022. Further refinement for article type was performed by including only original articles, excluding all other categories. Finally, the Web of Science categories of ‘veterinary science’, ‘plant science’ and ‘zoology’ were excluded. This returned over 800,000 publications with high specificity (~96.8%) for articles relevant to human health.

Rayyan.ai was used to export the data and screen for duplicates. Finally, the data was imported to Microsoft Excel by year to manually correct for wrong publication dates. The results were extracted for each country based on the affiliations of any listed author. Finally, we also recorded additional information including total population, gross domestic product in US dollars, GDP per capita in USD and World Bank income classification, as of 2022. These were obtained from publicly available World Bank, United Nations or national data sources or predictions made based on previous years.

Our methods are summarised graphically below;


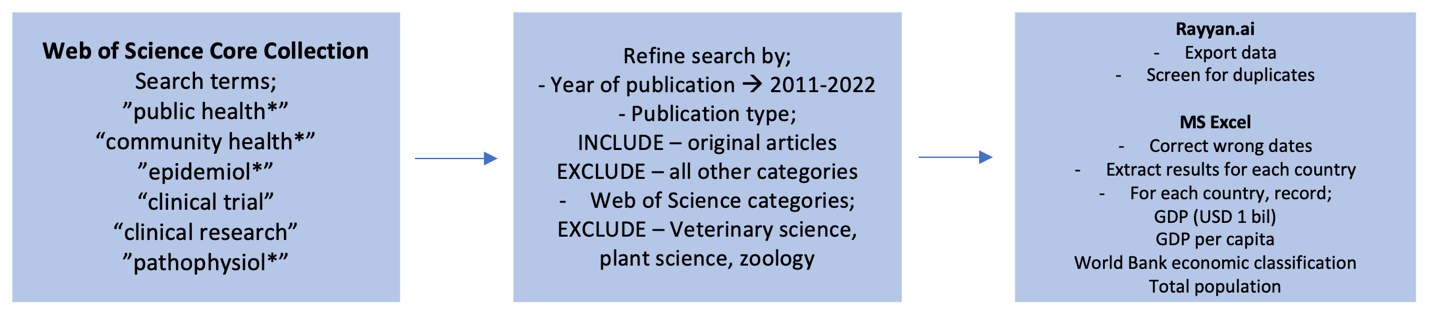


Very small countries (population < 1 million)

| **Country** | **Output** | **Population** | **Income classification** |
| --- | --- | --- | --- |
| Iceland | 989 | 381900 | High income |
| Luxembourg | 691 | 650774 | High income |
| Malta | 368 | 523417 | High income |
| Fiji | 211 | 929766 | Upper middle income |
| Montenegro | 131 | 616159 | Upper middle income |
| Grenada | 123 | 125438 | Upper middle income |
| Barbados | 116 | 281635 | High income |
| Bhutan | 106 | 782455 | Lower middle income |
| Brunei | 80 | 449002 | High income |
| St Kitts and Nevis | 78 | 47657 | High income |
| Monaco | 75 | 36469 | High income |
| Solomon Islands | 72 | 724273 | Lower middle income |
| Greenland | 63 | 56661 | High income |
| Suriname | 61 | 618040 | Upper middle income |
| Liechtenstein | 49 | 39327 | High income |
| Samoa | 45 | 222382 | Lower middle income |
| Micronesia | 43 | 114164 | Lower middle income |
| Seychelles | 42 | 100060 | High income |
| Guyana | 40 | 808726 | High income |
| Vanuatu | 39 | 326740 | Lower middle income |
| Cape Verde | 34 | 593149 | Lower middle income |
| Bahamas | 22 | 409984 | High income |
| Kiribati | 20 | 131232 | Lower middle income |
| Belize | 20 | 405272 | Upper middle income |
| Dominica | 20 | 72737 | Upper middle income |
| Maldives | 18 | 523787 | Upper middle income |
| Comoros | 16 | 836774 | Lower middle income |
| Palau | 16 | 18055 | Upper middle income |
| Sao Tome and Principe | 15 | 227380 | Lower middle income |
| San Marino | 15 | 33660 | High income |
| Andorra | 14 | 79824 | High income |
| Antigua and Barbuda | 10 | 93763 | High income |
| St Lucia | 8 | 179857 | Upper middle income |
| Marshall Island | 7 | 41569 | Upper middle income |
| Tonga | 7 | 106858 | Upper middle income |
| Nauru | 6 | 12668 | High income |
| Cook Islands | 4 | 17011 | Unclassified |
| St Vincent and the Grenadines | 4 | 103948 | Upper middle income |
| Tuvalu | 2 | 11312 | Upper middle income |
| Niue | 1 | 1934 | Unclassified |

Countries ranked by total output excluding very small countries and HICs

| **Country** | **Total output (2011-2022)** |
| --- | --- |
| China | 90212 |
| Brazil | 35289 |
| India | 26136 |
| Iran | 20041 |
| Turkey | 13832 |
| South Africa | 10751 |
| Mexico | 7498 |
| Egypt | 6729 |
| Thailand | 6409 |
| Pakistan | 5640 |
| Russia | 5392 |
| Argentina | 4801 |
| Ethiopia | 4742 |
| Malaysia | 4678 |
| Colombia | 4581 |
| Nigeria | 4502 |
| Kenya | 3480 |
| Uganda | 3061 |
| Bangladesh | 2893 |
| Indonesia | 2735 |
| Ghana | 2525 |
| Vietnam | 2348 |
| Serbia | 2199 |
| Peru | 2144 |
| Tanzania | 2025 |
| Tunisia | 1608 |
| Lebanon | 1592 |
| Nepal | 1429 |
| Jordan | 1395 |
| Cameroon | 1393 |
| Philippines | 1390 |
| Morocco | 1367 |
| Malawi | 1272 |
| Ecuador | 1158 |
| Ukraine | 1088 |
| Bulgaria | 1079 |
| Iraq | 1023 |
| Zambia | 1018 |
| Sri Lanka | 1006 |
| Senegal | 984 |
| Cuba | 975 |
| Burkina Faso | 892 |
| Venezuela | 867 |
| Zimbabwe | 838 |
| Mozambique | 767 |
| Sudan | 749 |
| Democratic Republic of Congo | 714 |
| Costa Rica | 648 |
| Rwanda | 641 |
| Cambodia | 640 |
| Algeria | 629 |
| Cote d’Ivoire | 557 |
| Mali | 503 |
| Kazakhstan | 479 |
| Benin | 467 |
| Gambia | 462 |
| Republic of Congo | 450 |
| Guatemala | 395 |
| Botswana | 394 |
| Bosnia | 387 |
| Madagascar | 376 |
| Lao PDR | 373 |
| Gabon | 333 |
| Myanmar | 324 |
| Georgia | 324 |
| Palestine | 324 |
| Sierra Leone | 323 |
| Yemen | 297 |
| Albania | 295 |
| Jamaica | 277 |
| Guinea | 270 |
| Paraguay | 250 |
| Papua New Guinea | 247 |
| Bolivia | 241 |
| Haiti | 241 |
| Macedonia | 236 |
| Belarus | 228 |
| Mongolia | 227 |
| Dominican Republic | 226 |
| Togo | 222 |
| Namibia | 218 |
| Afghanistan | 218 |
| Syria | 206 |
| Nicaragua | 199 |
| Honduras | 198 |
| Niger | 194 |
| Libya | 182 |
| Armenia | 175 |
| Liberia | 169 |
| Angola | 141 |
| Moldova | 125 |
| El Salvador | 124 |
| Kyrgyzstan | 120 |
| Central African Republic | 116 |
| Eswatini | 106 |
| Azerbaijan | 104 |
| Chad | 102 |
| Uzbekistan | 98 |
| Kosovo | 97 |
| Guinea Bissau | 85 |
| Burundi | 82 |
| Mauritius | 82 |
| Mauritania | 76 |
| Somalia | 64 |
| Lesotho | 62 |
| Eritrea | 42 |
| Tajikistan | 33 |
| Timor-Leste | 25 |
| Djibouti | 23 |
| Equatorial Guinea | 22 |
| North Korea | 8 |
| Turkmenistan | 5 |

Countries ranked by total output per capita, with World Bank Income Classification

| **Country** | **Income classification** |
| --- | --- |
| Serbia | Upper middle income |
| Lebanon | Lower middle income |
| Iran | Lower middle income |
| South Africa | Upper middle income |
| Gambia | Low income |
| Bulgaria | Upper middle income |
| Brazil | Upper middle income |
| Turkey | Upper middle income |
| Botswana | Upper middle income |
| Gabon | Upper middle income |
| Malaysia | Upper middle income |
| Tunisia | Lower middle income |
| Costa Rica | Upper middle income |
| Jordan | Lower middle income |
| Bosnia | Upper middle income |
| Macedonia | Upper middle income |
| Albania | Upper middle income |
| Argentina | Upper middle income |
| Jamaica | Upper middle income |
| Thailand | Upper middle income |
| Colombia | Upper middle income |
| Eswatini | Lower middle income |
| Georgia | Upper middle income |
| Cuba | Upper middle income |
| Namibia | Upper middle income |
| Ghana | Lower middle income |
| Republic of Congo | Lower middle income |
| Mongolia | Lower middle income |
| Mauritius | Upper middle income |
| Uganda | Low income |
| Kenya | Lower middle income |
| Ecuador | Upper middle income |
| China | Upper middle income |
| Peru | Upper middle income |
| Armenia | Upper middle income |
| Malawi | Low income |
| Palestine | Unclassified |
| Egypt | Lower middle income |
| Mexico | Upper middle income |
| Senegal | Lower middle income |
| Kosovo | Upper middle income |
| Zimbabwe | Lower middle income |
| Zambia | Lower middle income |
| Cameroon | Lower middle income |
| Laos | Lower middle income |
| Moldova | Upper middle income |
| Nepal | Lower middle income |
| Rwanda | Low income |
| Sri Lanka | Lower middle income |
| Guinea Bissau | Low income |
| Burkina Faso | Low income |
| Ethiopia | Low income |
| Cambodia | Lower middle income |
| Russia | Upper middle income |
| Sierra Leone | Low income |
| Paraguay | Upper middle income |
| Morocco | Lower middle income |
| Benin | Lower middle income |
| Liberia | Low income |
| Tanzania | Lower middle income |
| Venezuela | Upper middle income |
| Nicaragua | Lower middle income |
| Ukraine | Lower middle income |
| Lesotho | Lower middle income |
| Libya | Upper middle income |
| Togo | Low income |
| Belarus | Upper middle income |
| Kazakhstan | Upper middle income |
| Papua New Guinea | Lower middle income |
| Pakistan | Lower middle income |
| Vietnam | Lower middle income |
| Mozambique | Low income |
| Iraq | Upper middle income |
| Guatemala | Upper middle income |
| Mali | Low income |
| Haiti | Lower middle income |
| Central African Republic | Low income |
| Nigeria | Lower middle income |
| Djibouti | Lower middle income |
| Dominican Republic | Upper middle income |
| Cote d’Ivoire | Lower middle income |
| Bolivia | Lower middle income |
| El Salvador | Upper middle income |
| Guinea | Lower middle income |
| Honduras | Lower middle income |
| Timor-Leste | Lower middle income |
| India | Lower middle income |
| Kyrgyzstan | Lower middle income |
| Bangladesh | Lower middle income |
| Mauritania | Lower middle income |
| Sudan | Low income |
| Algeria | Lower middle income |
| Equatorial Guinea | Upper middle income |
| Madagascar | Low income |
| Philippines | Lower middle income |
| Eritrea | Low income |
| Azerbaijan | Upper middle income |
| Indonesia | Upper middle income |
| Syria | Low income |
| Yemen | Low income |
| Niger | Low income |
| Democratic Republic of Congo | Low income |
| Burundi | Low income |
| Myanmar | Lower middle income |
| Chad | Low income |
| Afghanistan | Low income |
| Angola | Lower middle income |
| Somalia | Low income |
| Tajikistan | Lower middle income |
| Uzbekistan | Lower middle income |
| Turkmenistan | Upper middle income |
| North Korea | Low income |

.
